# Antiviral Effect of Selenomethionine on Porcine Deltacoronavirus in Pig Kidney Epithelial Cells

**DOI:** 10.3389/fmicb.2022.846747

**Published:** 2022-02-15

**Authors:** Zhihua Ren, Guilin Jia, Hongyi He, Ting Ding, Yueru Yu, ZhiCai Zuo, Yanchun Hu, Zhijun Zhong, Shumin Yu, Huidan Deng, Liuhong Shen, Suizhong Cao, Guangneng Peng, Ya Wang, Dongjie Cai, Liping Gou, Xiaoping Ma, Haifeng Liu, Ziyao Zhou, Youtian Deng, Dingyong Yang, Junliang Deng

**Affiliations:** ^1^Key Laboratory of Animal Disease and Human Health of Sichuan Province, College of Veterinary Medicine, Sichuan Agricultural University, Chengdu, China; ^2^College of Animal Husbandry and Veterinary Medicine, Chengdu Agricultural College, Chengdu, China

**Keywords:** porcine deltacoronavirus, antiviral activities, antioxidant, selenomethionine, innate immunity

## Abstract

Porcine deltacoronavirus (PDCoV) is an emerging porcine intestinal coronavirus in recent years, which mainly causes different degrees of vomiting and diarrhea in piglets and has caused great harm to the swine husbandry worldwide since its report. Selenium is an essential trace element for organisms and has been demonstrated to have antiviral effects. In this study, pig kidney epithelial (LLC-PK) cells were used to study the antiviral activity of selenomethionine (Se-Met) (2, 4, 8, and 16 μM) against PDCoV by detecting the replication of the virus, the expression of the mitochondrial antiviral signal protein (MAVS) protein, and the phosphorylation of interferon regulatory factor-3 (IRF-3), IFN-α, and IFN-β, and the changes in glutathione content, glutathione peroxidase, superoxide dismutase activity, and hydrogen peroxide content in the cells. The results showed that Se-Met at higher than physiological concentrations (16 μM) could significantly inhibit the replication of PDCoV in LLC-PK cells and enhance the expression of MAVS protein and the phosphorylation of IRF-3. In addition, Se-Met also improved the intracellular production of IFNα/β and antioxidant capacity with increasing doses. These data suggest that the availability of selenium through selenomethionine supports the antiviral response in porcine kidney cells, and the specific mechanism is attributed to the improved cellular antioxidant capacity and activation of the MAVS pathway by Se-Met.

## Introduction

Coronaviruses are single-stranded positive capsular RNA viruses that mainly infect mammals and birds (Niederwerder and Hesse, 2018). According to the differences of genetic characteristics and serology, it can be divided into four genera: Alphacoronavirus, Betacoronavirus, Gamacoronavirus, and Deltacoronavirus (Ma et al., 2015). Among them, PDCoV belongs to the genus Deltacoronavirus in the Coronaviridae family and is a newly discovered porcine enteropathogenic coronavirus, which is mainly characterized by digestive system symptoms, such as diarrhea, dehydration, and varying degrees of vomiting in piglets (Xu et al., 2018; Yin et al., 2020). PDCoV was first reported in Hong Kong in 2012 and is now a pandemic worldwide (Zhang, 2016). The outbreak of PDCoV has caused serious economic losses to swine farming, but there is no widely used drug and vaccine in production. Therefore, we need to find a drug or nutrient with an antiviral effect commonly used in production to fight PDCoV infection.

As an essential nutrient element, selenium mainly exists in organic and inorganic selenium. Selenoprotein and selenoamino acids are the most common organic selenium, and selenomethionine (Se-Met) is the most common selenium form ingested by the organisms from food (Weekley and Harris, 2013). The antioxidant and immunomodulatory functions of selenium are most important, and these functions are mainly exerted by selenoproteins, such as glutathione peroxidase (GSH-PX) and thioredoxin reductase (TrxR). GSH-PX 1-4 is involved in hydrogen peroxide (H_2_O_2_) signal transduction and maintaining the cellular redox state (Brigelius-Flohe and Maiorino, 2013). In addition, GPX1, GPX4, and TrxR1 are also the most abundant selenoproteins in a variety of immune cells, and they play an important role in T cell proliferation and NK cell activation (Chu et al., 1992; Wingler and Brigelius-Flohe, 1999; Lei et al., 2007; Huang et al., 2012; Ingold et al., 2018).

Innate immunity acts as the first line of defense against pathogenic microorganisms. RIG-I-like receptor (RLR) is a member of innate immunity, which plays a significant role against RNA viruses (Ren et al., 2020). RLR recognizes the virus and can activate the mitochondrial antiviral signal protein (MAVS) and interferon regulatory factor (IRF), which in turn secrete interferon (IFN) to achieve antiviral effects (Hou et al., 2011). Currently, selenium has been found to have antiviral effects, including against coxsackie virus, influenza virus, human immunodeficiency virus (HIV), and porcine circovirus (PCV) in humans and animals (Beck et al., 1994; Schrauzer and Sacher, 1994; Nelson et al., 2001; Jaspers et al., 2007; Qian et al., 2018; Guillin et al., 2019; Bermano et al., 2021). The antiviral effect of selenium is mainly attributed to its antioxidant and immunomodulatory effects. For example, Se-Met (2, 4 mM) can inhibit PCV2 replication by inhibiting H_2_O_2_-mediated oxidative stress (Chen et al., 2012). In 450 HIV-1 seropositive patients, daily supplementation with 200 μg of yeast selenium not only decreased the viral load of HIV-1 but also increased the number of CD4^+^ T cells (Hurwitz et al., 2007).

Given the current situation of PDCoV, which is seriously harmful, there is no specific drug treatment. In this assay, we first examined the replication effect of Se-Met on PDCoV *in vitro*. Then, we further explored the potential mechanism of PDCoV inhibition by Se-Met from the perspective of innate immunity and anti-oxidation. It provides some theoretical basis and guiding significance for reducing PDCoV infection from antiviral nutrition.

## Materials and Methods

### Cells Culture and Reagents

Pig Kidney Epithelial (LLC-PK) cells were provided by Professor Zhanyong Wei of Henan Agricultural University. Cells were cultured in Minimum Essential Medium (MEM, Solarbio). MEM was supplemented with 8% fetal bovine serum (FBS, Gibco), 1% HEPES (Gibco), 100 IU/mL penicillin, and 100 IU/mL streptomycin solution. Se-Met was purchased from Sigma, United States. Referring to the results of Pan’s study (Pan et al., 2008), we confirmed that 16 μM Se-Met was not toxic to cells. Se-Met was diluted to 2, 4, 8, and 16 μM with MEM.

### Virus Stocks and Titration

PDCoV HNZK-04 strain (provided by Professor Zhanyong Wei, Henan Agricultural University) was used in this study. PDCoV was propagated in a maintenance medium (MEM supplemented with 1% antibiotics, 1% HEPES, and 5 μg/mL trypsin) containing LLC-PK cells. The number of infectious PDCoV particles was determined based on the 50% tissue culture infectious dose (TCID_50_) in LLC-PK cells, according to the method described by Zhai’s study (Zhai et al., 2019).

### Assays for Antiviral Activity of Selenomethionine

To understand the inhibitory activity of Se-Met (2, 4, 8, and 16 μM) on PDCoV, cells were added to six-well plates and allowed to grow to about 90%. The virus stock was diluted to 100 TCID_50_ and inoculated into cells for 1 h. The virus liquid was discarded, washed twice with D-Hanks, and Se-Met solution was added and cultured at 37°C for 24 h. The virus infection control group (group V) and the blank control group (group C) were randomly within the plates. At last, the viral load of each group was measured.

### Assays of Virus Titer

RNA from LLC-PK cells was extracted using TRIzol reagent (Invitrogen) and cDNA synthesis using cDNA Synthesis Super (TransGen Biotech) according to the manufacturer’s instructions. Then, RT-qPCR was performed using Perfect Start™ Green qPCR Super (TransGen Biotech). The virus was determined by absolute RT qPCR, and the primers for the PDCoV M gene are shown in Table 1. The 2^–ΔΔ*CT*^ method was used to differentiate between control and treated cells.

**TABLE 1:** Primer sequence.

Primer	Sequence (5′→3′)	Bp
M	F: CGCGTAATCGTGTGATCTATGT R: CCGGCCTTTGAAGTGGTTAT	
IFN-α	F: CTTCTGGACCTGGTTGCCC R: GCTCCTGGCACAAATGAGGA	103
IFN-β	F: GGAGATTATGCAACCACCA R: CCAGCCAGTGCTAGAGAAA	112
β-actin	F: CTGCGGCATCCACGAAACT R: AGGGCCGTGATCTCCTTCTG	147

### Assays of Interferon

According to the kit instructions (Jiangsu MEIMIAN Co., Ltd. China), expression of IFN-α and IFN-β was determined using ELISA and RT qPCR. Primers for RT qPCR are shown in Table 1, and β-actin was used as a reference gene (Chen et al., 2012). There are three independent replicates done for treatment.

### Detection of Oxidative Stress and Antioxidant Indicators

The cell pellet was collected at the end of cell culture after washing and centrifugation. One milliliter of the extract was added to the cell pellet, and the cells were disrupted by sonication for subsequent testing. The contents of glutathione (GSH) and H_2_O_2_, as well as the activities of GSH-PX and superoxide dismutase (SOD), were measured using kits (Nanjing Jiancheng Bioengineering Institute, China). There are three independent replicates were done for treatment.

### Western Blot Analysis

The expression of MAVS protein and the phosphorylation of IRF-3 in innate immunity was examined using Western blot to determine whether Se-Met affects innate immunity. The following primary antibodies were used: Anti-Phospho-IRF-3 (Ser396), Monoclonal Antibody (MA5-14947) (Invitrogen Corporation, NY, United States), Anti-IRF-3 Polyclonal antibody (11312-1-AP), and Anti-MAVS antibody (14341-1-AP) (Proteintech Group, Wuhan, China).

### Statistical Analysis

Results were expressed as the means ± standard deviation (SD). The test data were analyzed for the significance of difference by one-way ANOVA using SPSS 26 (*P* < 0.05).

## Results

### Selenomethionine Has Antiviral Activity on Porcine Deltacoronavirus

**FIGURE 1:**
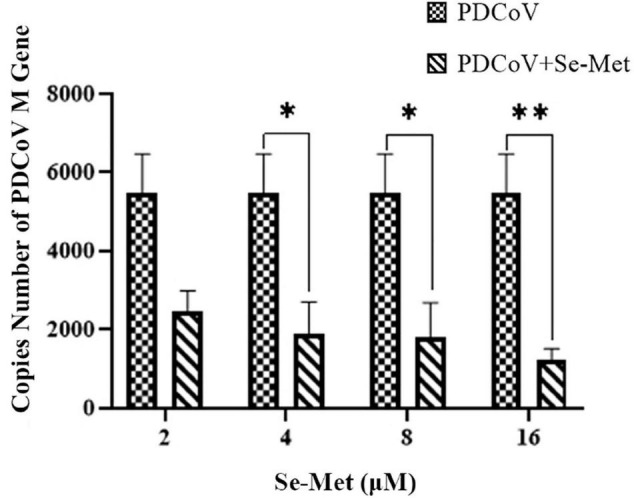
Antiviral effect of Se-Met against PDCoV. LLC-PK cells were incubated with PDCoV for 1 h, and 2, 4, 8, and 16 μM of Se-Met was added. *0.01 < *P* < 0.05, ^**^P < 0.01.

LLC-PK cells were treated with different concentrations of Se-Met (2, 4, 8, and 16 μM) after inoculation with PDCoV. The copy number of the PDCoV M gene in each group was detected using RT qPCR 24 h after virus infection. The results are shown in Figure 1. The 4 and 8 μM Se-Met could significantly inhibit the copy number of virus M gene (0.01 < *P* < 0.05). Moreover, after the virus was treated with 16 μM Se-Met, the replication was extremely decreased (*P* < 0.01). In conclusion, Se-Met can inhibit the replication of PDCoV in a dose-dependent manner, with 16 μM Se-Met having the best effect.

### Selenomethionine Can Enhance Cellular Immunity After Porcine Deltacoronavirus Infection

**FIGURE 2:**
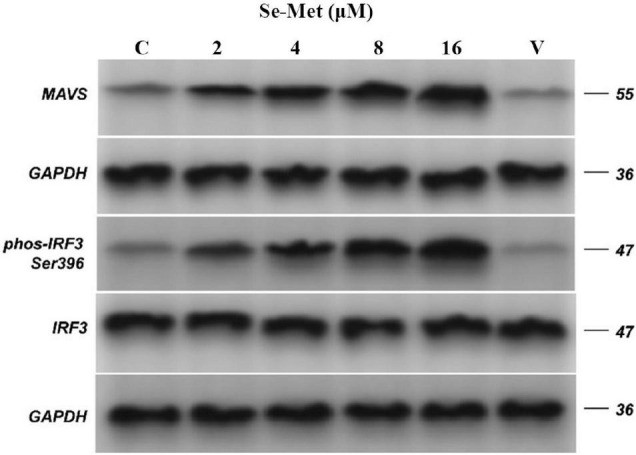
Effects of Set-Met on the expression of MAVS protein and the phosphorylation of IRF-3 induced by PDCoV. LLC-PK cells were incubated with PDCoV for 1 h, and 2, 4, 8, and 16 μM of Se-Met was added. The expression of MAVS protein and the phosphorylation of IRF-3 were detected by western blot. Results were mean ± SD for three individual experiments.

**FIGURE 3:**
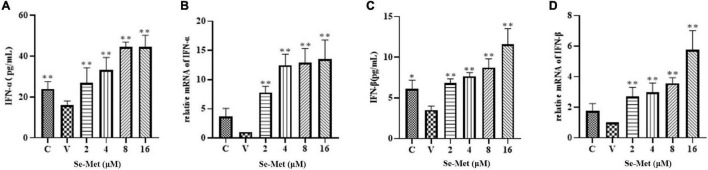
Effects of Set-Met on the changes of IFN-α/β in cells induced by PDCoV. LLC-PK cells were incubated with PDCoV for 1 h, and 2, 4, 8, and 16 μM of Se-Met was added. Changes in IFN-α/β were detected by ELISA **(A,C)** and RT qPCR **(B,D)**. *0.01 < *P* < 0.05, ^**^P < 0.01.

Evasion of innate immunity has emerged as a way for the virus to maintain replication. We treated LLC-PK cells with Se-Met in four concentrations to further investigate whether Se-Met inhibits viral replication by improving cellular immunity. First, we used Western blot to detect the expression of MAVS protein and phosphorylation of IRF-3 intracellularly and then used ELISA and RT-qPCR to detect the changes of IFN-α and IFN-β. Western blot results showed that PDCoV was able to significantly reduce the protein expression of MAVS and the phosphorylation of IRF-3 (0.01 < *P* < 0.05). After Se-Met treatment, the protein expression of MAVS and the phosphorylation of IRF-3 were significantly increased in all concentration groups (*P* < 0.01, Figure 2). We can see from Figure 3 that all concentrations of Se-Met significantly increased the production of IFNα/β in cells compared with group V (*P* < 0.01).

### Selenomethionine Can Enhance the Antioxidant Capacity of Cells After Porcine Deltacoronavirus Infection

**FIGURE 4:**
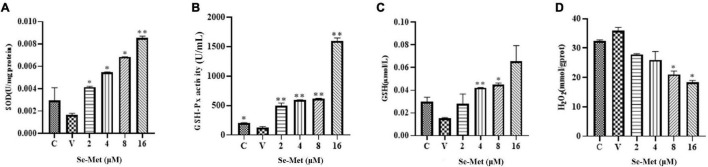
Effects of Set-Met on the changes of oxidative/antioxidant indexes in cells induced by PDCoV. LLC-PK cells were incubated with PDCoV for 1 h, and 2, 4, 8, and 16 μM of Se-Met was added. **(A)** Changes in SOD. **(B)** Changes in GSH-PX. **(C)** Changes in GSH. **(D)** Changes in H_2_O_2_. *0.01 < *P* < 0.05, ^**^P < 0.01.

The effect of Set-Met on oxidative/antioxidant factors of LLC-PK cells induced by PDCoV. Se-Met and virus were applied to LLC-PK cells using the modalities described above. After cytocentrifugation, cells were disrupted with ultrasound and used to detect GSH-Px, H_2_O_2_, SOD, and GSH. As shown in Figure 4, PDCoV was able to reduce the activity of GSH-PX in the cells significantly (0.01 < *P* < 0.05), and although the contents of SOD and GSH were also decreased, they did not change significantly (*P* > 0.5). We found that 16 μM Se-Met was able to significantly increase the activity of GSH-PX and the content of SOD (*P* < 0.01). After PDCoV was treated with 8 and 16 μM Se-Met, the H_2_O_2_ content was significantly reduced (0.01 < *P* < 0.05). In summary, PDCoV induces oxidative stress in cells; however, Se-Met can alleviate this damage.

## Discussion

Since PDCoV was first reported in 2012, PDCoV infections have been reported in regions every year, and new lineages have been slowly discovered. PDCoV is currently seriously damaging the development of the pig industry, but there are no commercial vaccines and antiviral drugs commonly used in the breeding industry. To develop a good drug against PDCoV infection, we found that the nutrient selenium has biological functions such as antiviral, antioxidant, and immunomodulatory (Guillin et al., 2019; Bermano et al., 2021). Whether Se-Met, as an organoselenium, has an inhibitory effect on the infection of PDCoV is unknown. In this experiment, we first determined that the TCID_50_ of PDCoV for LLC-PK cells was 10^–4⋅15^/0.1 mL. Subsequently, referring to the results of Pan’s study (Pan et al., 2008), we confirmed that 16 μM Se-Met did not affect the growth of cells, which was used as the maximum effective concentration of Se-Met in this experiment for the determination of subsequent antiviral and antioxidant assays.

Given the current situation that there is no specific drug treatment for PDCoV, some people have successively explored the potential of broad-spectrum antiviral drugs in treating PDCoV. For example, lithium chloride and diammonium glycyrrhizinate could inhibit PDCoV replication in LLC-PK cells in a dose-dependent manner (Zhai et al., 2019). In addition, Zhang explored the inhibitory effect of LJ001 on PDCoV in three ways (Zhang et al., 2020). He found that the antiviral effect of pretreatment was not significant, which may be because the receptor sites available for virus attachment on the cell membrane are altered after the cells are pretreated with the drug, which affects the fusion of virus and cells (Zhang et al., 2020). Studies on the inhibition of viral replication by selenium have been reported, for example, HIV, Coxsackie virus, influenza virus, and PCV (Beck et al., 1994; Schrauzer and Sacher, 1994; Nelson et al., 2001; Jaspers et al., 2007; Qian et al., 2018). In this experiment, PDCoV was treated with Se-Met in a post-treatment manner. The results showed that Se-Met significantly inhibits PDCoV replication, and 16 μM Se-Met has the best effect; 16 μM Se-Met showed better antiviral activity, but 16 μM was far higher than the normal physiological concentration, and too high concentration of selenium would be counterproductive (Sivertsen et al., 2007; Zhao et al., 2016). We found that Se-Met at 8 and 16 μM had similar antiviral activity, and combined with the dangers of high concentrations of selenium, we believe that Se-Met at 8 μM is in line with clinical application. However, if we want to treat PDCoV, 8 μM Se-Met will not necessarily be clinically effective, so we also need to combine *in vivo* experiments.

After virus infection, the oxidative stress state of the organism can destroy the immune system in the body, which in turn facilitates virus replication (Zhang Z. et al., 2019). GSH-PX is an important enzyme in the biological function of selenium, and it achieves antioxidant effects by scavenging peroxides in the body (Tian et al., 2021). It was previously reported that selenium could alleviate virus-induced oxidative stress. For example, in the PK-15 cell model of PCV infection, 6 μM Se-Met can inhibit the increase of PCV2 replication by enhancing the activity of GSH-PX1 and inhibiting the production of H_2_O_2_ (Chen et al., 2012). SOD is an important antioxidant enzyme in organisms that scavenges superoxide radicals. Styblo et al. (2007) found that after mice were infected with the influenza virus, selenium supplementation significantly increased the SOD activity of the mouse liver. In addition, oseltamivir is an effective antiviral drug. Nano-selenium is surface-modified by oseltamivir, which significantly inhibits ROS generation induced by H1N1 (Li et al., 2017). This experiment verified that Se-Met could inhibit the replication of PDCoV in LLC-PK cells, and the GSH-Px, H_2_O_2_, GSH, and SOD changes in the virus groups and the treatment groups were further compared. The experimental results are consistent with previous results on other viruses; that is, PDCoV inhibits the viability of cellular GSH-Px, increasing the content of H_2_O_2_ in the cell, which in turn causes oxidative stress. The addition of Se-Met can significantly increase the ability of GSH-PX and GSH in cells. From this, we can infer that Se-Met can alleviate the oxidative stress caused by PDCoV by improving the antioxidant capacity of cells, thereby achieving the effect of inhibiting virus replication.

During virus infection and replication, innate immunity acts as the first line of defense of the immune response, clearing the virus from the host. As a key receptor for the recognition of RNA viruses, RLR can activate the downstream MAVS after binding to the virus, which in turn stimulates the expression of IRF and nuclear factor kappa-B (Chen et al., 2018; Ren et al., 2020). Then, the release of IFN, a key cytokine for the host to see viral immunity, is stimulated by IRF. However, viruses have been able to evade or fight the host’s immune system in various ways in continuous evolution (Wang et al., 2016; Kouwaki et al., 2021). It has been found that Hepatitis B can competitively bind MAVS with the help of lactate and further interfere with the binding of RLR to MAVS to achieve the effect of evading innate immunity (Zhang W. et al., 2019). Then, At least eight proteins encoded by severe acute respiratory syndrome coronavirus have been identified as interferon antagonists (Devaraj et al., 2007; Kopecky-Bromberg et al., 2007; Wathelet et al., 2007; Siu et al., 2009). In addition, PDCoV was also found to evade host immune responses, and it not only avoided IFN-β activation but also inhibited IFN-β production induced by SeV or Poly (I: C) (Luo et al., 2016). This reason may be attributed to the degradation of interferon by the ubiquitin proteasome encoded by PDCoV (Ji et al., 2020). Based on previous studies, we hope to improve the antiviral effect of host innate immunity by adding Se-Met. As expected, Se-Met could significantly increase the expression of MAVS protein, the phosphorylation of IRF-3, and the mRNA level of IFN-α/β. Therefore, we conclude that Se-Met can activate MAVS and IRF-3 in innate immunity and secrete a series of IFN to inhibit PDCoV replication.

## Conclusion

In summary, this study suggests that Se-Met could inhibit PDCoV replication in a dose-dependent manner. The underlying mechanism may be attributed to the activation of MAVS and IRF-3 in innate immunity by Se-Met, which in turn secretes a series of cytokines. In addition, it may also be because Se-Met can improve the antioxidant capacity of cells.

## Data Availability Statement

The original contributions presented in the study are included in the article/supplementary material, further inquiries can be directed to the corresponding author/s.

## Author Contributions

ZR, ZCZ, JD, YH, ZJZ, and SY contributed to the conception and design of the study. TD, HH, and YY performed the statistical analysis. GJ wrote the first draft of the manuscript. The rest of the authors reviewed and revised the manuscript. All authors reviewed the manuscript, read, and approved the submitted version.

## Conflict of Interest

The authors declare that the research was conducted in the absence of any commercial or financial relationships that could be construed as a potential conflict of interest.

## Publisher’s Note

All claims expressed in this article are solely those of the authors and do not necessarily represent those of their affiliated organizations, or those of the publisher, the editors and the reviewers. Any product that may be evaluated in this article, or claim that may be made by its manufacturer, is not guaranteed or endorsed by the publisher.

## Abbreviations

GSH: glutathione
GSH-PX: glutathione peroxidase
HIV: human immunodeficiency virus
H_2_O_2_: hydrogen peroxide
IRF-3: interferon regulatory factor-3
IFN: interferon
LLC-PK: Pig Kidney Epithelial
MAVS: mitochondrial antiviral signal protein
PDCoV: Porcine deltacoronavirus
PCV: Porcine circovirus
RLR: RIG-I-like receptor
Se-Met: selenomethionine
SOD: superoxide dismutase
TrxR: thioredoxin reductase
TCID_50_: tissue culture infectious dose.

## References

[B1] Beck M. A. Kolbeck P. C. Rohr L. H. Shi Q. Morris V. C. Levander O. A. (1994). Benign human enterovirus becomes virulent in selenium-deficient mice. *J. Med. Virol.* 43 166–170. 10.1002/jmv.1890430213 8083665

[B2] Bermano G. Meplan C. Mercer D. K. Hesketh J. E. (2021). Selenium and viral infection: are there lessons for COVID-19? *Br. J. Nutr.* 125 618–627. 10.1017/S0007114520003128 32758306 PMC7503044

[B3] Brigelius-Flohe R. Maiorino M. (2013). Glutathione peroxidases. *Biochim. Biophys. Acta* 1830 3289–3303. 10.1016/j.bbagen.2012.11.020 23201771

[B4] Chen X. Liu S. Goraya M. U. Maarouf M. Huang S. Chen J. L. (2018). Host Immune Response to Influenza A Virus Infection. *Front. Immunol.* 9:320. 10.3389/fimmu.2018.00320 29556226 PMC5845129

[B5] Chen X. Ren F. Hesketh J. Shi X. Li J. Gan F. (2012). Selenium blocks porcine circovirus type 2 replication promotion induced by oxidative stress by improving GPx1 expression. *Free Radic. Biol. Med.* 53 395–405. 10.1016/j.freeradbiomed.2012.04.035 22580339

[B6] Chu F. F. Esworthy R. S. Doroshow J. H. Doan K. Liu X. F. (1992). Expression of plasma glutathione peroxidase in human liver in addition to kidney, heart, lung, and breast in humans and rodents. *Blood* 79 3233–3238. 1339300

[B7] Devaraj S. G. Wang N. Chen Z. Chen Z. Tseng M. Barretto N. (2007). Regulation of IRF-3-dependent innate immunity by the papain-like protease domain of the severe acute respiratory syndrome coronavirus. *J. Biol. Chem.* 282 32208–32221. 10.1074/jbc.M704870200 17761676 PMC2756044

[B8] Guillin O. M. Vindry C. Ohlmann T. Chavatte L. (2019). Selenium. *Selenoproteins and Viral Infection*. *Nutrients* 11:2101. 10.3390/nu11092101 31487871 PMC6769590

[B9] Hou F. Sun L. Zheng H. Skaug B. Jiang Q. X. Chen Z. J. (2011). MAVS forms functional prion-like aggregates to activate and propagate antiviral innate immune response. *Cell* 146 448–461. 10.1016/j.cell.2011.06.041 21782231 PMC3179916

[B10] Huang Z. Rose A. H. Hoffmann P. R. (2012). The role of selenium in inflammation and immunity: from molecular mechanisms to therapeutic opportunities. *Antioxid. Redox Signal* 16 705–743. 10.1089/ars.2011.4145 21955027 PMC3277928

[B11] Hurwitz B. E. Klaus J. R. Llabre M. M. Gonzalez A. Lawrence P. J. (2007). Suppression of human immunodeficiency virus type 1 viral load with selenium supplementation: a randomized controlled trial. *Arch. Intern. Med.* 167 148–154. 10.1001/archinte.167.2.148 17242315

[B12] Ingold I. Berndt C. Schmitt S. Doll S. Poschmann G. (2018). Selenium Utilization by GPX4 Is Required to Prevent Hydroperoxide-Induced Ferroptosis. *Cell* 172 409-422. 10.1016/j.cell.2017.11.048 29290465

[B13] Jaspers I. Zhang W. Brighton L. E. Carson J. L. Styblo M. Beck M. A. (2007). Selenium deficiency alters epithelial cell morphology and responses to influenza. *Free Radic. Biol. Med.* 42 1826–1837. 10.1016/j.freeradbiomed.2007.03.017 17512462 PMC2048669

[B14] Ji L. Wang N. Ma J. Cheng Y. Wang H. Sun J. (2020). Porcine deltacoronavirus nucleocapsid protein species-specifically suppressed IRF7-induced type I interferon production via ubiquitin-proteasomal degradation pathway. *Vet. Microbiol.* 250:108853. 10.1016/j.vetmic.2020.108853 32992291 PMC7834071

[B15] Kopecky-Bromberg S. A. Martinez-Sobrido L. Frieman M. Baric R. A. Palese P. (2007). Severe acute respiratory syndrome coronavirus open reading frame (ORF) 3b, ORF 6, and nucleocapsid proteins function as interferon antagonists. *J. Virol.* 81 548–557. 10.1128/JVI.01782-06 17108024 PMC1797484

[B16] Kouwaki T. Nishimura T. Wang G. Oshiumi H. (2021). RIG-I-Like Receptor-Mediated Recognition of Viral Genomic RNA of Severe Acute Respiratory Syndrome Coronavirus-2 and Viral Escape From the Host Innate Immune Responses. *Front. Immunol.* 12:700926. 10.3389/fimmu.2021.700926 34249006 PMC8267574

[B17] Lei X. G. Cheng W. H. McClung J. P. (2007). Metabolic regulation and function of glutathione peroxidase-1. *Annu. Rev. Nutr.* 27 41–61. 10.1146/annurev.nutr.27.061406.093716 17465855

[B18] Li Y. Lin Z. Guo M. Xia Y. Zhao M. Wang C. (2017). Inhibitory activity of selenium nanoparticles functionalized with oseltamivir on H1N1 influenza virus. *Int. J. Nanomed.* 12 5733–5743. 10.2147/IJN.S140939 28848350 PMC5557909

[B19] Luo J. Fang L. Dong N. Fang P. Ding Z. Wang D. (2016). Porcine deltacoronavirus (PDCoV) infection suppresses RIG-I-mediated interferon-beta production. *Virology* 495 10–17. 10.1016/j.virol.2016.04.025 27152478 PMC7111668

[B20] Ma Y. Zhang Y. Liang X. Lou F. Oglesbee M. Krakowka S. (2015). Origin, evolution, and virulence of porcine deltacoronaviruses in the United States. *mBio* 6:e00064. 10.1128/mBio.00064-15 25759498 PMC4453528

[B21] Nelson H. K. Shi Q. Van Dael P. Schiffrin E. J. Blum S. Barclay D. (2001). Host nutritional selenium status as a driving force for influenza virus mutations. *FASEB. J.* 15 1727–1738. 11481250

[B22] Niederwerder M. C. Hesse R. A. (2018). Swine enteric coronavirus disease: a review of 4 years with porcine epidemic diarrhoea virus and porcine deltacoronavirus in the United States and Canada. *Transbound. Emerg. Dis.* 65 660–675. 10.1111/tbed.12823 29392870 PMC7169865

[B23] Pan Q. Huang K. He K. Lu F. (2008). Effect of different selenium sources and levels on porcine circovirus type 2 replication in vitro. *J. Trace Elem. Med. Biol.* 22 143–148. 10.1016/j.jtemb.2008.02.002 18565426

[B24] Qian G. Liu D. Hu J. Gan F. Hou L. Zhai N. (2018). SeMet attenuates OTA-induced PCV2 replication promotion by inhibiting autophagy by activating the AKT/mTOR signaling pathway. *Vet. Res.* 49:15. 10.1186/s13567-018-0508-z 29439710 PMC5812231

[B25] Ren Z. Ding T. Zuo Z. Xu Z. Deng J. Wei Z. (2020). Regulation of MAVS Expression and Signaling Function in the Antiviral Innate Immune Response. *Front. Immunol.* 11:1030. 10.3389/fimmu.2020.01030 32536927 PMC7267026

[B26] Schrauzer G. N. Sacher J. (1994). Selenium in the maintenance and therapy of HIV-infected patients. *Chem. Biol. Interact.* 91 199–205. 10.1016/0009-2797(94)90040-x 7514960

[B27] Siu K. L. Kok K. H. Ng M. J. Poon V. K. M. Yuen K. Y. Zheng B. J. (2009). Severe acute respiratory syndrome coronavirus M protein inhibits type I interferon production by impeding the formation of TRAF3.TANK.TBK1/IKKepsilon complex. *J. Biol. Chem.* 284 16202–16209. 10.1074/jbc.M109.008227 19380580 PMC2713514

[B28] Sivertsen T. Vie E. Bernhoft A. Baustad B. (2007). Vitamin E and selenium plasma concentrations in weanling pigs under field conditions in Norwegian pig herds. *Acta. Vet. Scand.* 49:1. 10.1186/1751-0147-49-1 17201915 PMC1779789

[B29] Styblo M. Walton F. S. Harmon A. W. Sheridan P. A. Beck M. A. (2007). Activation of superoxide dismutase in selenium-deficient mice infected with influenza virus. *J. Trace Elem. Med. Biol.* 21 52–62. 10.1016/j.jtemb.2006.11.001 17317526

[B30] Tian R. Geng Y. Yang Y. Seim I. Yang G. (2021). Oxidative stress drives divergent evolution of the glutathione peroxidase (GPX) gene family in mammals. *Integr. Zool.* 16 696–711. 10.1111/1749-4877.12521 33417299

[B31] Wang D. Fang L. Shi Y. Zhang H. Gao L. Peng G. (2016). Porcine Epidemic Diarrhea Virus 3C-Like Protease Regulates Its Interferon Antagonism by Cleaving NEMO. *J. Virol.* 90 2090–2101. 10.1128/JVI.02514-15 26656704 PMC4733996

[B32] Wathelet M. G. Orr M. Frieman M. B. Baric R. S. (2007). Severe acute respiratory syndrome coronavirus evades antiviral signaling: role of nsp1 and rational design of an attenuated strain. *J. Virol.* 81 11620–11633. 10.1128/JVI.00702-07 17715225 PMC2168762

[B33] Weekley C. M. Harris H. H. (2013). Which form is that? The importance of selenium speciation and metabolism in the prevention and treatment of disease. *Chem. Soc. Rev.* 42 8870–8894. 10.1039/c3cs60272a 24030774

[B34] Wingler K. Brigelius-Flohe R. (1999). Gastrointestinal glutathione peroxidase. *Biofactors* 10 245–249. 10.1002/biof.5520100223 10609889

[B35] Xu Z. Zhong H. Zhou Q. Du Y. Chen L. Zhang Y. (2018). A Highly Pathogenic Strain of Porcine Deltacoronavirus Caused Watery Diarrhea in Newborn Piglets. *Virol. Sin.* 33 131–141. 10.1007/s12250-018-0003-8 29569144 PMC6178105

[B36] Yin L. Chen J. Li L. Guo S. Xue M. Zhang J. (2020). Aminopeptidase N Expression, Not Interferon Responses, Determines the Intestinal Segmental Tropism of Porcine Deltacoronavirus. *J. Virol.* 94 e00480–20. 10.1128/JVI.00480-20 32376622 PMC7343211

[B37] Zhai X. Wang S. Zhu M. He W. Pan Z. Su S. (2019). Antiviral Effect of Lithium Chloride and Diammonium Glycyrrhizinate on Porcine Deltacoronavirus In Vitro. *Pathogens* 8:144. 10.3390/pathogens8030144 31505777 PMC6789623

[B38] Zhang J. (2016). Porcine deltacoronavirus: overview of infection dynamics, diagnostic methods, prevalence and genetic evolution. *Virus Res.* 226 71–84. 10.1016/j.virusres.2016.05.028 27270129 PMC7114555

[B39] Zhang W. Wang G. Xu Z. G. Tu H. Hu F. Dai J. (2019). Lactate Is a Natural Suppressor of RLR Signaling by Targeting MAVS. *Cell* 178 176–189. 10.1016/j.cell.2019.05.003 31155231 PMC6625351

[B40] Zhang Y. Xia L. Yuan Y. Li Q. Han L. Yang G. (2020). Rhodanine derivative LJ001 inhibits TGEV and PDCoV replication in vitro. *Virus Res.* 289:198167. 10.1016/j.virusres.2020.198167 32956749 PMC7501054

[B41] Zhang Z. Rong L. Li Y. P. (2019). Flaviviridae Viruses and Oxidative Stress: implications for Viral Pathogenesis. *Oxid. Med. Cell Longev.* 2019:1409582. 10.1155/2019/1409582 31531178 PMC6720866

[B42] Zhao Z. Barcus M. Kim J. Lum K. L. Mills C. Lei X. G. (2016). High Dietary Selenium Intake Alters Lipid Metabolism and Protein Synthesis in Liver and Muscle of Pigs. *J. Nutr.* 146 1625–1633. 10.3945/jn.116.229955 27466604 PMC4997278

